# Supplementary material for: Architecture and Function of Mechanosensitive Membrane Protein Lattices
Source: Sci Rep. 2016 Jan 14;6:19214. doi: 10.1038/srep19214 (PMC4725903; doi:10.1038/srep19214)
Supplement: Supplementary Information [file srep19214-s1.pdf]

Architecture and Function of Mechanosensitive Membrane Protein Lattices

Supplementary Information

Osman Kahraman[1], Peter D. Koch[2], William S. Klug[3], and Christoph A. Haselwandter[1]

[1]*Departments of Physics & Astronomy and Biological Sciences, University of Southern California, Los Angeles, CA 90089, USA*

[2]*Department of Systems Biology, Harvard Medical School, Boston, MA 02115, USA*

[3]*Department of Mechanical and Aerospace Engineering, University of California, Los Angeles, CA 90095, USA*

S1 Gradient of the bilayer thickness deformation field

To confirm that the lipid bilayer thickness deformations induced by MscL lattices are sufficiently small for the standard leading-order model in Eq. (1) of the main text to be valid, we computed the magnitude of the gradient of the bilayer thickness deformation field, $||\nabla u||$, for the systems of closed pentameric and tetrameric MscL shown in Fig. 1 of the main text, and also for the ground-state lattice architectures of closed pentameric and tetrameric MscL discussed in the main text (see Fig. S1). We find that, consistent with previous analytic and computational studies of gramicidin channels [1] and MscL [2], $||\nabla u|| \leq 0.16$ for all MscL arrangements considered. Indeed, the bilayer thickness deformation gradients are generally small in bilayer regions separating strongly interacting MscL, which is the regime most relevant for the ground-state lattices discussed in the main text. This can be understood by noting that, for the smallest MscL edge-to-edge separations allowed by steric constraints on lipid size, the separation between two adjacent MscL edges is of the same order as the elastic decay length of thickness deformations, $\ell_c = (a^2 K_b / K_t)^{1/4} \approx 1$ nm [3, 4]. Thickness deformations therefore decay only weakly between MscL in ground-state lattices, with an approximate maximum gradient ≈ 0.02 (see bottom panels of Fig. S1).

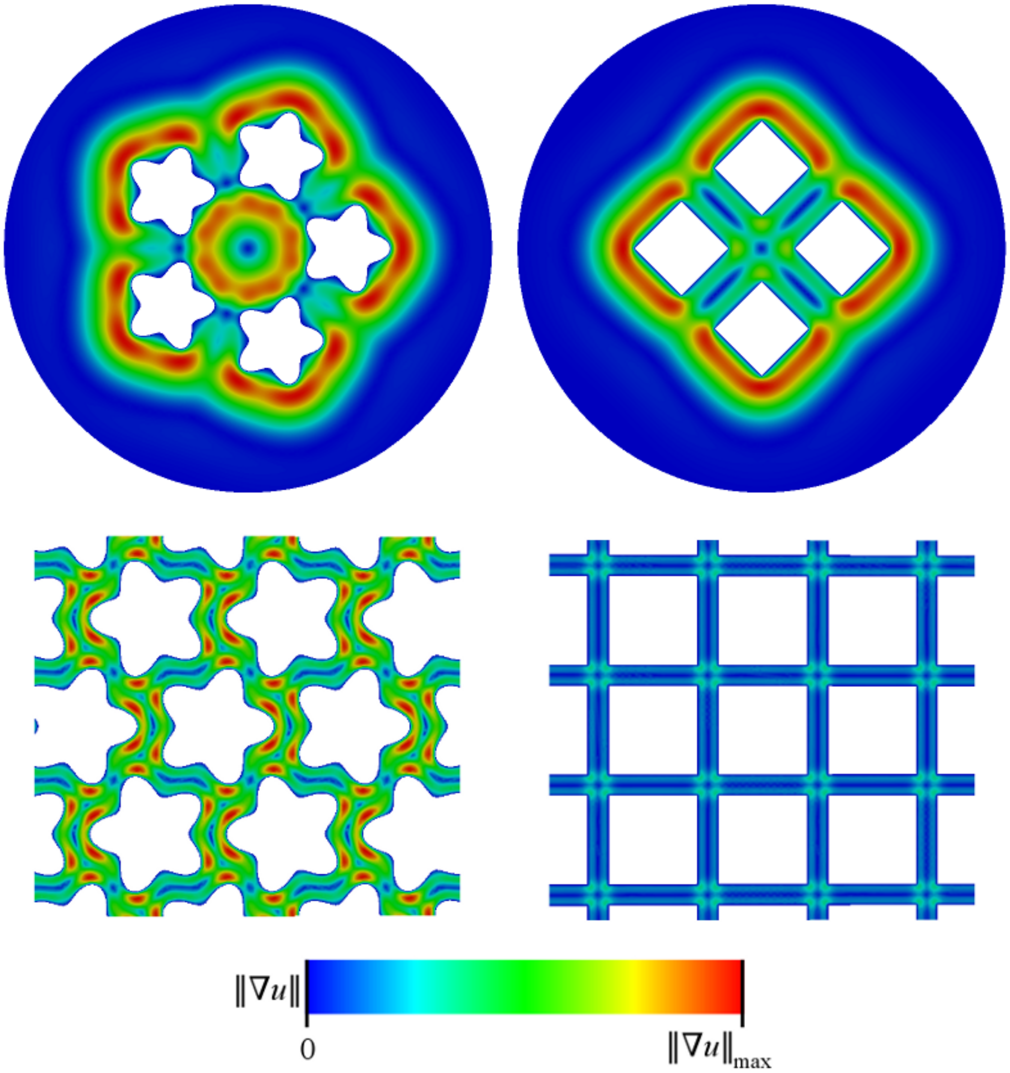

Figure S1: The magnitude $||\nabla u||$ of the gradient of MscL-induced thickness deformations u for the systems of closed pentameric and tetrameric MscL shown in Fig. 1 of the main text (top panels with color scale $||\nabla u||_{\max} = 0.156$), and the ground-state lattice architectures of closed pentameric and tetrameric MscL discussed in the main text (bottom panels with color scale $||\nabla u||_{\max} = 0.016$).

S2 Pairwise additivity

In Fig. 2 of the main text we study pairwise additivity of thickness-mediated interactions by varying the MscL center-to-center distance d. To supplement these results, we consider here variations in MscL orientation in a system of three MscL (see right inset in Fig. S2). In particular, we fix the relative separation of two MscL at the steric cutoff d_{st} and let the third MscL revolve around the first MscL, gradually approaching the second MscL. We then compare three-body interaction energies to the corresponding energies obtained by pairwise summation for the cylinder model of MscL [2,4–6], tetrameric MscL, pentameric MscL, and a system composed of one tetrameric and two pentameric MscL. In agreement with Fig. 2 of the main text, we find that the qualitative trends obtained by summation over pairwise interaction energies are similar to that obtained from the full three-body interactions, but that non-pairwise contributions to thickness-mediated interactions can be $> 1k_BT$ in magnitude and, depending on protein shape and orientation, increase as well as decrease the interaction energy (see left inset in Fig. S2).

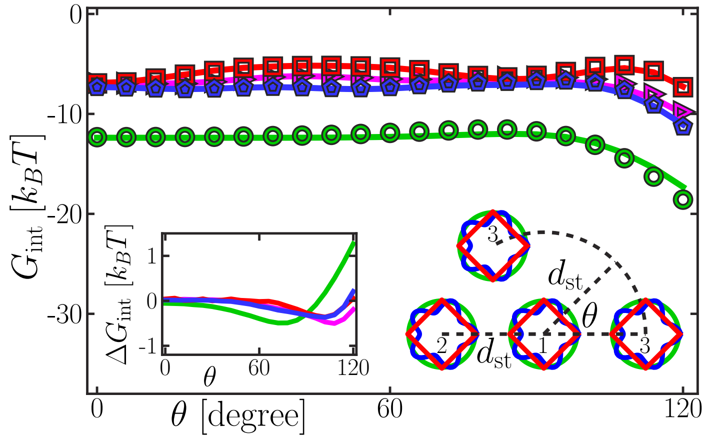

Figure S2: Pairwise and non-pairwise thickness-mediated interaction energies as in Fig. 2 of the main text but for variations in MscL orientation. We consider a three-body system in which the relative separation of MscL 1 and 2 is kept fixed at the steric cutoff $d_{\rm st}$ (right inset). MscL 3 is kept at a constant separation $d_{\rm st}$ from MscL 1 but, starting from $\theta = 0$, rotates around MscL 1 to $\theta = 120^\circ$, thereby approaching MscL 2. In the final configuration, all three MscL are at a separation $d_{\rm st}$ from each other. Green, red, blue, and purple curves show three-body interaction energies per MscL for cylinders, tetramers, pentamers, and a system consisting of one tetramer (MscL 2) and two pentamers (MscL 1 and 2). Circles, squares, pentagons, and triangles indicate the corresponding interaction energies obtained by pairwise summation. The left inset shows the energy difference per MscL between the three-body interaction energies and the corresponding interaction energies obtained by summation over pairwise interactions.

S3 Lattices of tetrameric MscL

We calculated the interaction energies for infinite honeycomb, square, and hexagonal MscL lattices for the cylinder model of MscL [2, 4–6] (see Fig. S3) and tetrameric MscL (see Fig. S4) using appropriate unit cells with dimensions which vary with lattice spacing. The symmetries of infinite lattices are imposed by applying zero Neumann boundary conditions along the boundaries of the unit cells.

Following the finite element procedure outlined in the Methods section of the main text, we used the unit cells in Figs. S3 and S4 to calculate $G_{\rm int}$ for infinite MscL lattices (see Fig. 3 of the main text). To gain further insight into pairwise additivity of thickness-mediated interactions, we compare here these interaction energies with the corresponding interaction energies obtained by summation over pairwise interactions within a radius of 15 nm about each MscL (see Fig. S5). For the cylinder model of MscL we find that, irrespective of whether multi-body or only pairwise interactions are considered, the honeycomb lattice is preferred at large d and the hexagonal lattice with $d = d_{\rm st}$ provides the ground-state lattice architecture (see Fig. S5(a)). For tetrameric MscL (see Fig. S5(b)), multi-body and pairwise interactions also yield the same basic picture for the competition between different lattice architectures, with one important exception: the competition between infinite face-on square and infinite shifted square lattices.

In particular, pairwise interactions suggest that the face-on square lattice has a lower ground-state energy than the shifted square lattice even for infinite lattice sizes (Fig. S5(b)), while our multi-body calculations show that infinite face-on square and infinite shifted square lattices have the same ground-state energy within numerical accuracy (Fig. S5(b) and Fig. 3 of the main text). (For finite lattice sizes, pairwise and multi-body interactions both yield the face-on square lattice as the ground-state lattice architecture of tetrameric MscL (Fig. 3 of the main text).) Thus, the assumption of pairwise additivity breaks down for the special case of the competition between infinite face-on square and infinite shifted square lattices of tetrameric MscL. This can be understood by noting that face-on square and shifted square lattices of tetrameric MscL have the same packing fraction while, in the ground state, the typical edge-to-edge separation of tetrameric MscL in face-on square and shifted square lattices is comparable to the elastic decay length of thickness deformations, $\ell_c = (a^2 K_b/K_t)^{1/4} \approx 1$ nm [3, 4]. As a result, one expects that, in the limit of small MscL separations, the two lattice architectures have very similar thickness deformation footprints and thickness deformation energies, which is in agreement with our multi-body calculations. The pairwise approximation fails to account for this special feature of infinite lattices of tetrameric MscL because the center-to-center distances of neighboring

tetrameric MscL in shifted square lattices are inhomogeneous which, in the pairwise approximation, leads to overestimation of the thickness deformation energy associated with shifted square lattices.

In the main text we focus on large clusters of tetrameric MscL (see Fig. 3 of the main text). For completeness, we consider here the opposite limit of very small clusters of tetrameric MscL. In particular, we compare the multi-body lattice energies of a honeycomb lattice constructed from 24 tetrameric MscL, square and shifted square lattices constructed from 16 MscL, and a hexagonal lattice constructed from 19 MscL (see Fig. S6). The sizes of honeycomb, square, and hexagonal lattices are thereby chosen so as to yield the respective (translational) lattice symmetries under global lattice rotations. Comparison between finite and infinite (Fig. 3 of the main text and Fig. S5(b)) lattice energies shows that cluster size can strongly affect the magnitude of the lattice energy per MscL, but does not affect the competition between square, honeycomb, and hexagonal lattice architectures. As in Fig. 3 of the main text, we find that the face-on square lattice with $d = d_{\mathrm{st}}$ provides the ground-state lattice architecture for finite lattices, with the hexagonal and tip-on square lattices yielding favorable lattice symmetries at larger (fixed) d.

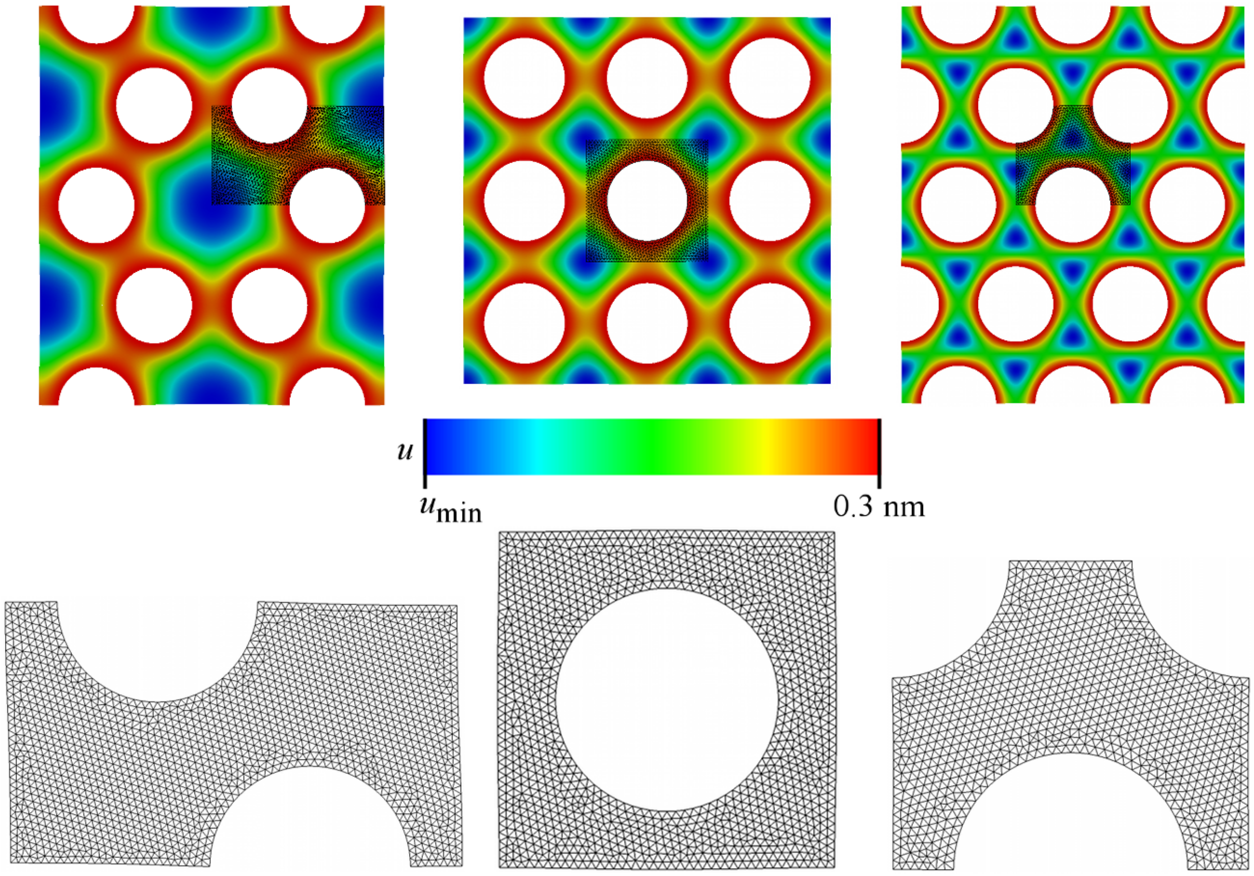

Figure S3: Unit cells used for infinite lattice calculations for the cylinder model of MscL [2,4–6] for honeycomb (left panels), square (middle panels), and hexagonal (right panels) lattices. In the first row of panels, the meshes used in our finite element calculations are shown as black triangulated patches superimposed on the calculated thickness deformation fields. (Thickness deformations ranging from $u = U_c = 0.3$ nm (red) to $u = u_{\mathrm{min}}$ (blue), with $u_{\mathrm{min}} = -0.04$, 0.09, and 0.23 nm for honeycomb, square, and hexagonal lattices with $d = 7$ nm, respectively.) The second row shows enlarged versions of the finite element meshes. We adjust the meshing with the lattice spacing d to ensure good numerical accuracy. Along the unit cell boundaries the normal projections of the gradients of u are zero by symmetry.

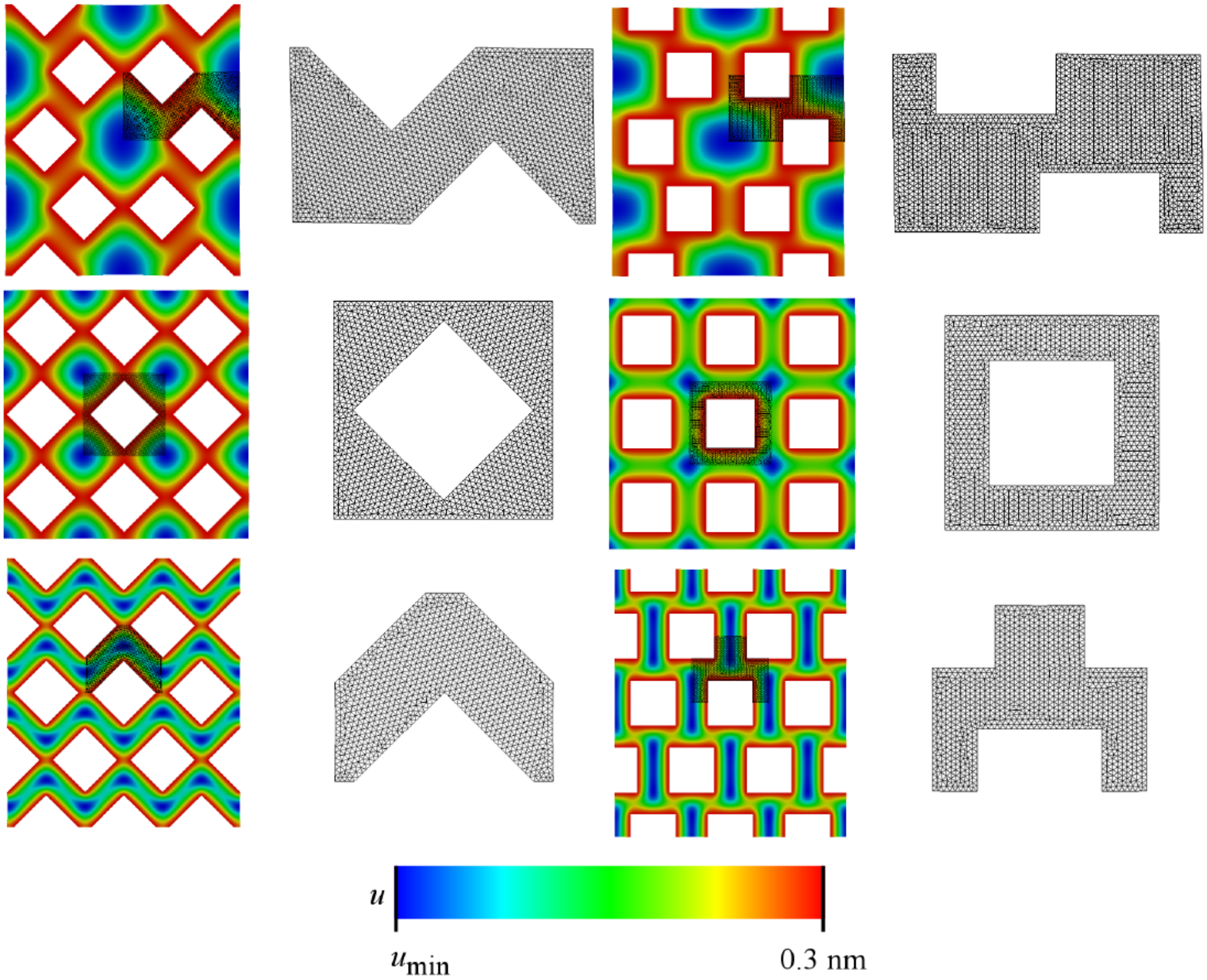

Figure S4: Unit cells used for the infinite lattice calculations for tetrameric MscL in Fig. 3 of the main text for tip-on and face-on honeycomb (top row of panels), square (middle row of panels), and hexagonal (bottom row of panels) lattices. (Thickness deformations ranging from $u = U_c = 0.3$ nm (red) to $u = u_{\min}$ (blue), with $u_{\min} = -0.04$, 0.24, 0.17, and 0.06 nm for honeycomb, hexagonal, face-on square, and tip-on square lattices with $d = 7$ nm, respectively.) See the caption of Fig. S3 for further details.

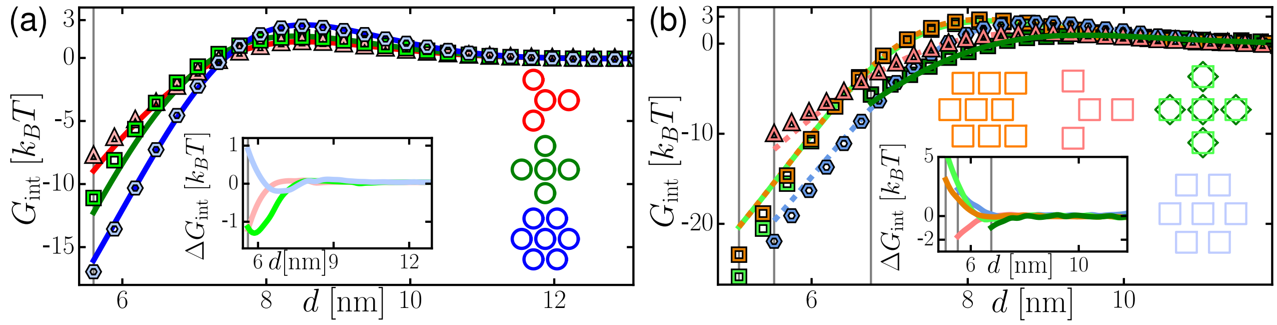

Figure S5: Pairwise additivity of thickness-mediated interactions in MscL lattices. Thickness-mediated interaction energy per closed MscL for (a) the cylinder model of MscL [2, 4–6] and (b) tetrameric MscL in infinite honeycomb, square, and hexagonal lattices as a function of lattice spacing. Solid and dashed curves are obtained using the unit cells in Figs. S3 and S4, and triangles, squares, and hexagons indicate the corresponding interaction energies in honeycomb, square, and hexagonal lattices obtained by summation over pairwise interactions within a radius of 15 nm about each MscL. In (a), red, green, and blue curves correspond to honeycomb, square, and hexagonal lattices (see insets). In (b), red and blue curves indicate face-on honeycomb and hexagonal lattices, and tip-on, face-on, and shifted square lattices correspond to green, light green, and orange curves (see insets). The insets show the difference between the multi-body energies and the corresponding energies obtained from pairwise interactions. Vertical lines indicate $d = d_{\rm st}$.

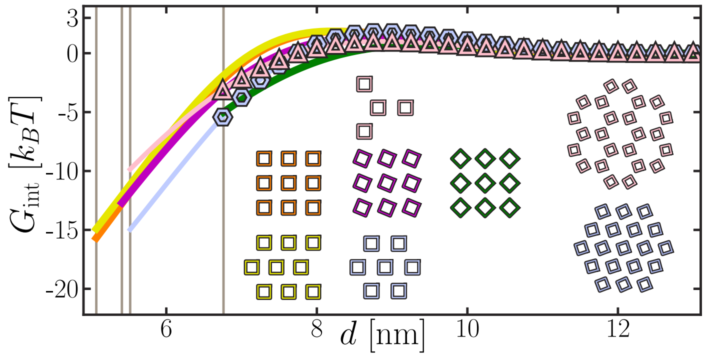

Figure S6: Lattice architecture of small clusters of tetrameric MscL. Thickness-mediated interaction energy per closed tetrameric MscL for small honeycomb, square, hexagonal, and shifted square lattices composed of ≈ 20 MscL as a function of lattice spacing. The solid curves correspond to the lattice architectures in the left insets as indicated by the color coding. Triangles and hexagons correspond to the orientational arrangements shown as far-right insets, which are optimized at each d through Monte Carlo simulations with simulated annealing of pair interaction potentials as in Fig. 4(a) of the main text. Vertical lines indicate steric cutoffs on MscL separations.

S4 Lattices of pentameric MscL

In Fig. 4 of the main text we consider finite honeycomb, square, and hexagonal lattices of pentameric MscL composed of ≈ 220 MscL, which are shown in Fig. S7 (regular lattices) and Fig. S8 (distorted lattices [7]).

In analogy to the small clusters of tetrameric MscL in Fig. S6, we calculated bilayer thickness-mediated interactions in small honeycomb, square, and hexagonal lattices of pentameric MscL composed of 24, 16, and 19 MscL, respectively (see Fig. S9(a)). As for the larger clusters considered in Fig. 4(a) of the main text, we find that the hexagonal (honeycomb) lattice is preferred at small (large) d. The same qualitative conclusions are obtained by summation over pairwise interactions within a radius of 15 nm about each MscL in infinite lattices. In analogy to Fig. 4(b) of the main text, we also calculated interaction energies in small distorted lattices [7] (see Fig. S9(b)). As in Fig. 4 of the main text, we find that, at the largest ϕ allowed by steric constraints, an MscL arrangement similar to the closest packed of the distorted lattices (bottom-left lattice in Fig. S9(b)), which corresponds to a distorted hexagonal lattice with alternate rows of MscL aligned in opposite directions, provides the ground-state lattice architecture, and that, in disperse clusters with variable d, face-on orientation of three neighbouring MscL (top-right lattice in Fig. S9(b)) yields a favorable lattice architecture for a range of ϕ.

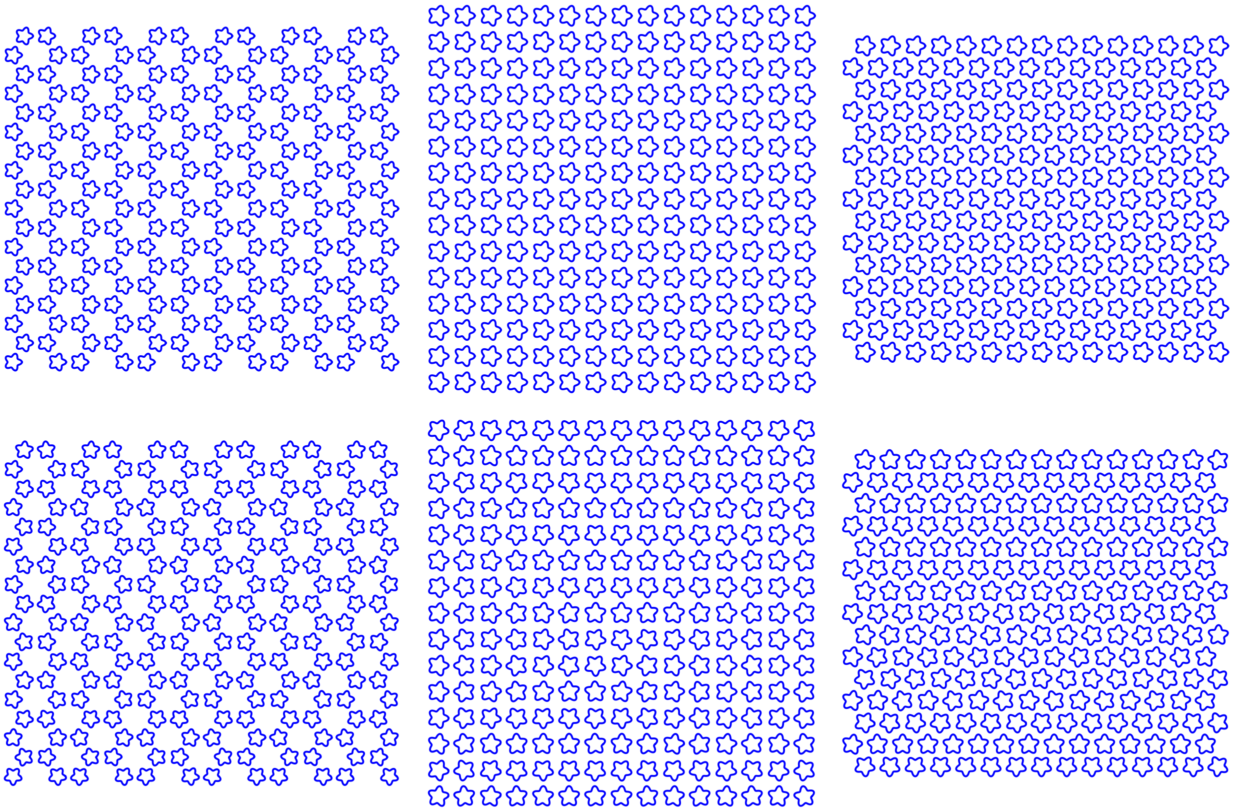

Figure S7: Pentameric MscL lattices used for Fig. 4(a,b) of the main text. From left to right: honeycomb (216 MscL), square (225 MscL), and hexagonal (225 MscL) lattices. In the lower panels, MscL orientations are optimized at $d = 7$ nm through Monte Carlo simulations with simulated annealing of pair interaction potentials.

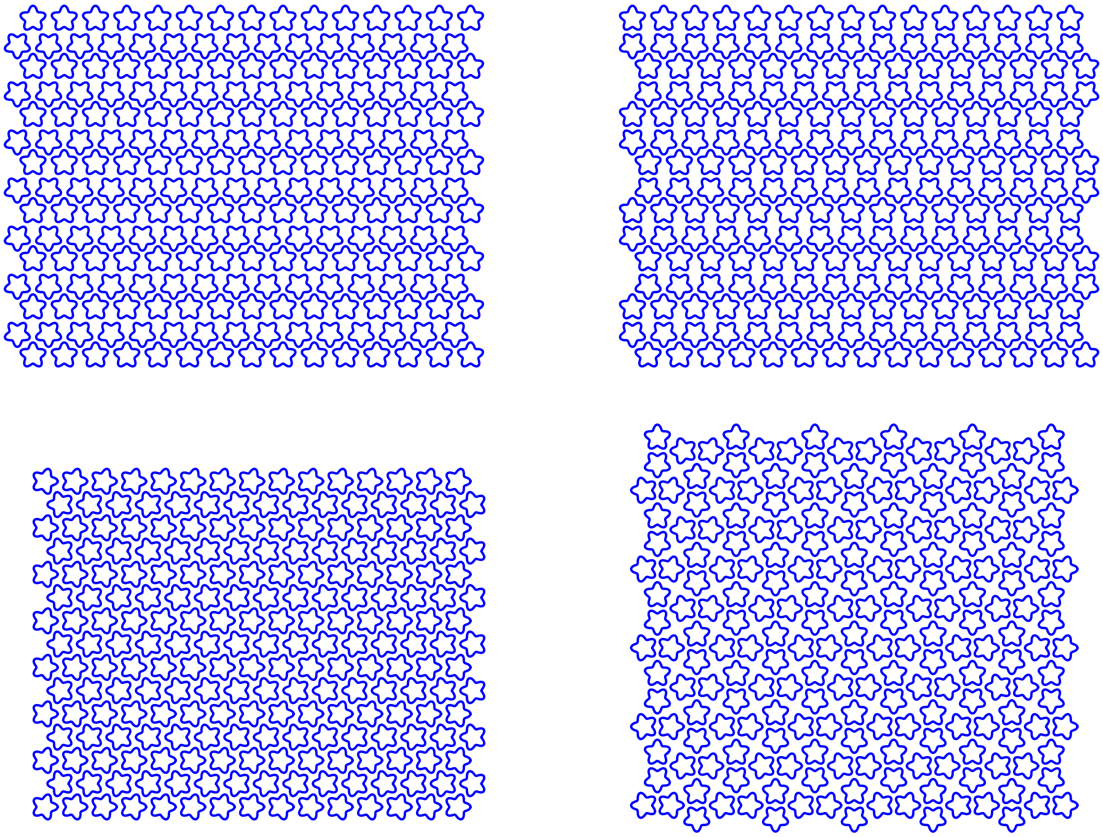

Figure S8: Distorted lattices [7] of pentameric MscL used for Fig. 4(b) of the main text. All distorted lattices were constructed from 225 pentameric MscL with the exception of the bottom-right lattice, which was constructed from 220 MscL.

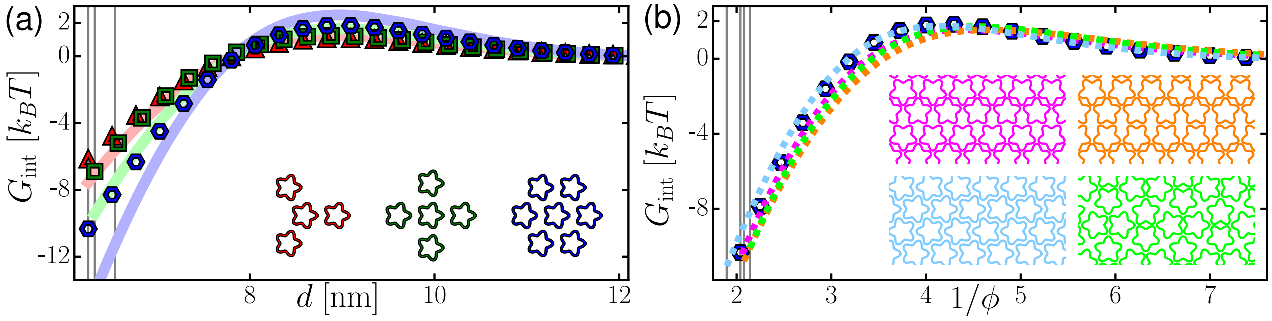

Figure S9: Lattice architecture of small clusters of pentameric MscL. Thickness-mediated interaction energy per closed pentameric MscL for small clusters composed of ≈ 20 MscL. In (a), triangles, squares, and hexagons correspond to honeycomb (24 MscL), square (16 MscL), and hexagonal (19 MscL) lattices, respectively. For comparison, thick curves indicate the corresponding interaction energies obtained for infinite lattices by summation over pairwise interactions within a radius of 15 nm about each MscL. (b) Interaction energy per pentameric MscL as a function of inverse area packing fraction for distorted lattices [7]. We constructed the purple, orange, cyan, and green distorted lattice architectures from 19, 18, 16, and 24 MscL, respectively. Blue hexagons correspond to the hexagonal lattice in (a). Area packing fraction is calculated as the ratio of MscL area to the area of the lattice unit cell.

S5 Simulated annealing of MscL clusters

To supplement Fig. 5 of the main text, we show in Fig. S10 additional data on the ground-state lattice architectures of MscL obtained, as in Fig. 5 of the main text, by Monte Carlo simulations of translational and rotational diffusion of MscL with simulated annealing of pair potentials, but using different random seeds or initial conditions. Our conclusions in the main text regarding the preferred lattice architectures of tetrameric and pentameric MscL are not sensitive to the details of the simulated annealing Monte Carlo simulations.

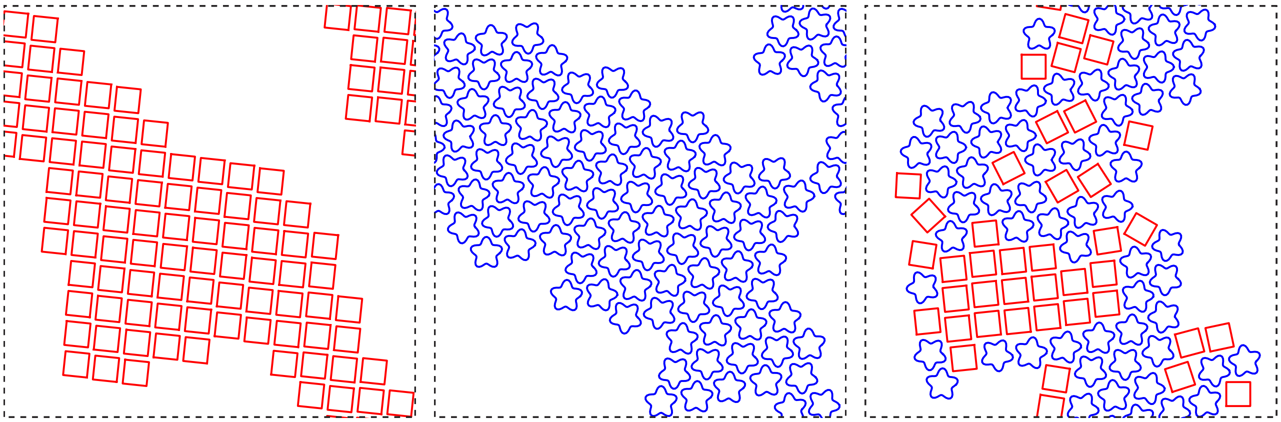

Figure S10: Spontaneous ordering of closed MscL obtained through Monte Carlo simulations of translational and rotational diffusion with simulated annealing of pair interaction potentials as in Fig. 5 of the main text, but using different random seeds or initial conditions. We used periodic boundary conditions with 100 MscL and, for the right-most panel, 40 tetrameric and 60 pentameric MscL.

To confirm the predictions in Figs. 3 and 4 of the main text regarding the minimum-energy lattice architectures of disperse MscL clusters with fixed minimum d, we repeated our Monte Carlo simulations of translational and rotational diffusion of MscL with simulated annealing of pair potentials using an isotropically restricted minimum d. We find that, for a minimum $d = 6$ nm, tetrameric MscL form hexagonal clusters (Fig. S11(a)) with alternating rows of approximately face-on and tip-on orientations. The energy of this mixed face-on and tip-on hexagonal arrangement of tetrameric MscL at $d = 6$ nm is within $< 0.2 k_B T$ per MscL of the energy of the corresponding tip-on and face-on hexagonal lattices, respectively. When we increase the steric cutoff to a larger value of $d = 8$ nm, tetrameric MscL form tip-on square lattices (Fig. S11(b)). Furthermore, at an intermediate value of $d = 7$ nm, we observe the coexistence of hexagonal and square lattice architectures for tetrameric MscL (Fig. S12(a)). If the same isotropic cutoff of $d = 7$ nm is applied to pentameric MscL we obtain a hexagonal lattice (Fig. S12(b)). These results are in excellent agreement with our predictions in Figs. 3 and 4 of the main text.

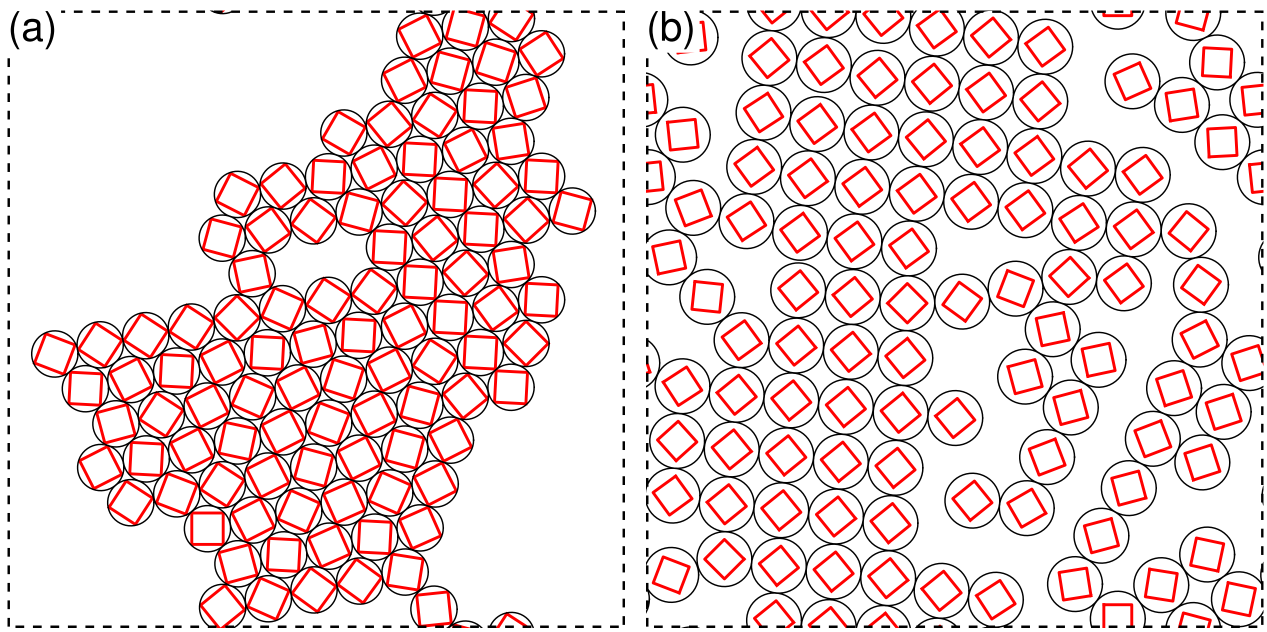

Figure S11: Ordering of closed tetrameric MscL obtained through Monte Carlo simulations of translational and rotational diffusion of MscL with simulated annealing of pair interaction potentials using an isotropically restricted minimum d (shown as black circles). We used the minimum separations (a) $d = 6$ nm and (b) 8 nm. See also Supplementary Videos S6 and S7.

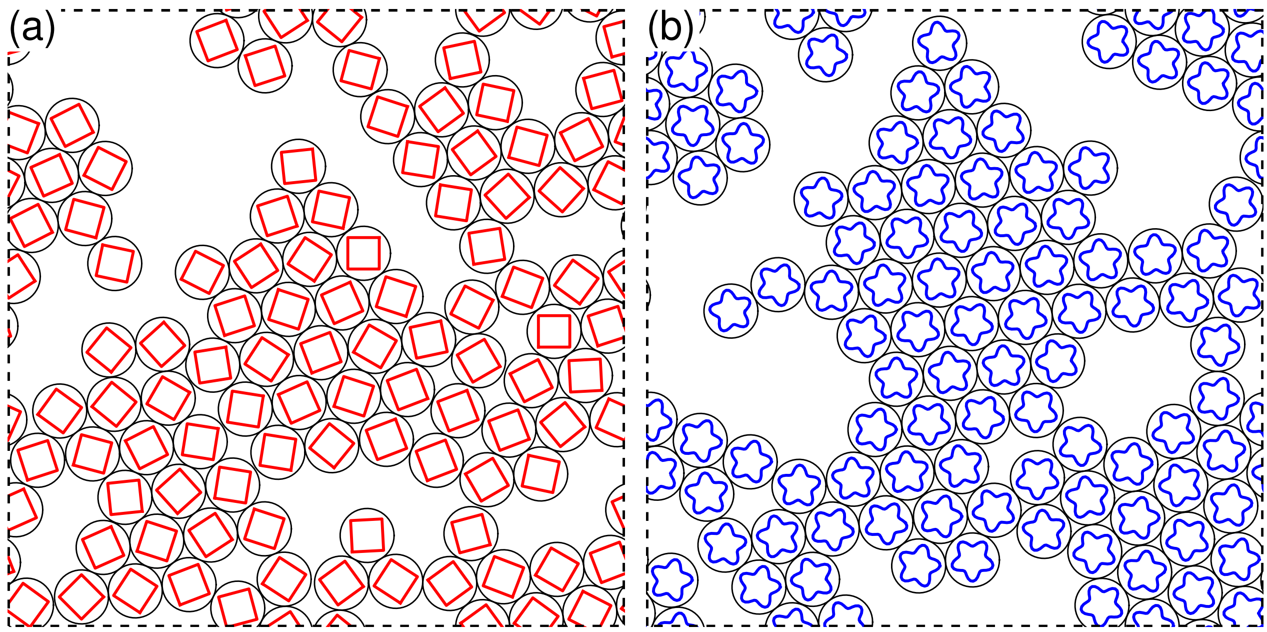

Figure S12: Ordering of closed (a) tetrameric and (b) pentameric MscL obtained through Monte Carlo simulations of translational and rotational diffusion of MscL with simulated annealing of pair interaction potentials using an isotropically restricted minimum $d = 7$ nm (shown as black circles). See also Supplementary Videos S8 and S9.

S6 MscL clusters at finite temperature

A comprehensive theoretical and computational study of the effects of thermal perturbations on the MscL lattice architectures studied here would require collective-move Monte Carlo simulations of translational and rotational MscL diffusion [8], combined with suitable models of the hydrodynamics of the lipid bilayer [9], which is beyond the scope of the present work. However, to provide a simple estimate of the extent to which thermal fluctuations might compete with, and "smear out," thickness-mediated ordering of MscL, we implemented standard Monte Carlo simulations at room temperature (see Fig. S13 for typical snapshots from these simulations). In these room-temperature Monte Carlo simulations, individual MscL are allowed to carry out translational and rotational Monte Carlo moves according to the standard Metropolis algorithm, which we implemented following the steps described in the Methods section of the main text.

Based on these simple Monte Carlo simulations we find that, for the typical MscL cluster sizes observed experimentally and considered here, face-on square lattices of tetrameric MscL only distort slightly due to thermal fluctuations (left panel in Fig. S13). For clusters of pentameric MscL we find that, at room temperature, the approximately hexagonal translational ordering of pentameric MscL is preserved, but thermal fluctuations decrease the local orientational ordering of pentameric MscL (middle panel in Fig. S13). This can be understood from Figs. 3 and 4 of the main text, which show that the predicted orientational ordering of MscL is strongly favored for tetrameric MscL but only weakly favored for pentameric MscL due to frustration of directional interactions. The lattice architecture of mixed clusters of tetrameric and pentameric MscL obtained from our room-temperature Monte Carlo simulations (right panel in Fig. S13) is similar to the lattice architecture obtained at zero temperature, in which orientational ordering of pentameric MscL is also distorted to accommodate tetrameric MscL lattices.

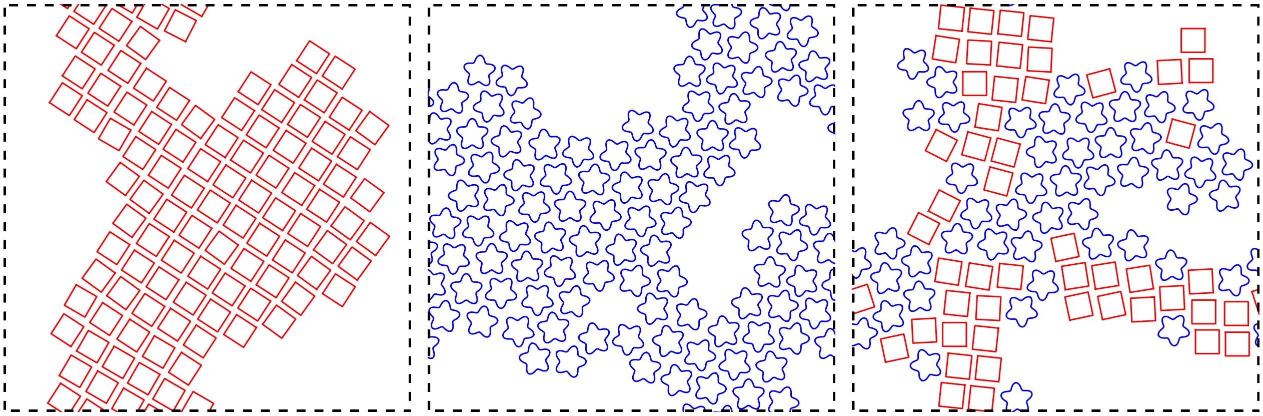

Figure S13: Clustering of closed MscL obtained through Monte Carlo simulations of translational and rotational diffusion at constant temperature $T = T_{\rm rm}$, where $T_{\rm rm} = 298$ K is the room temperature. We used periodic boundary conditions with 100 MscL and, for the right-most panel, 50 tetrameric and 50 pentameric MscL.

S7 Activation of MscL lattices

MscL gating in the interior of a densely packed MscL cluster would require [10] an energetically costly reorganization of the MscL cluster to a less densely packed lattice architecture, so as to accommodate the increased size of open MscL. In contrast, MscL localized at the corners of MscL lattices are subject to weaker interactions and, hence, have a lower activation barrier. To calculate the lattice activation barriers in Fig. 6(a) of the main text we therefore considered the energy required to displace a tetrameric or pentameric MscL located at the corners of the respective ground-state lattices, to a position sufficiently removed from other MscL to sterically allow opening of MscL (see Fig. S14).

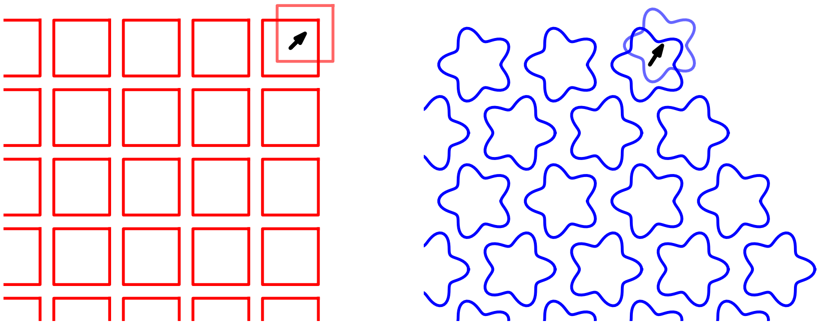

Figure S14: To estimate the activation barriers of tetrameric and pentameric MscL lattices in Fig. 6(a) of the main text we calculated the energy required to displace MscL at the corners of the respective ground-state lattices to the closest positions which sterically allow MscL in the open state (displacements indicated by arrows). For tetrameric (left panel) and pentameric (right panel) MscL, the ground-state lattice architectures correspond to the face-on square lattice and the distorted hexagonal lattice [7] shown in the bottom-left inset of Fig. 4(b) of the main text, respectively.

To supplement Fig. 6(b,c) of the main text, we show in Fig. S15 additional data on the ground-state lattice architectures of mixed clusters of closed and open MscL obtained, as in Fig. 6(b,c) of the main text, through Monte Carlo simulations of translational and rotational diffusion of MscL with simulated annealing of pair potentials, but using different random seeds or initial conditions. Our conclusions in the main text regarding the preferred lattice architectures of partially activated MscL clusters are not sensitive to the details of the simulated annealing Monte Carlo simulations. Further insights regarding the lattice architectures of partially activated MscL clusters can be obtained by idealizing the interface between two sub-clusters of closed and open MscL as being approximately straight (see Fig. S16(a)), and calculating the total thickness-mediated interaction energy $\bar{G}_{\text{int}}$ of the two sub-clusters (see Fig. S16(b,c)). We find that, at close separations (small values of the sub-cluster separation w), interactions are unfavorable for lattices of tetrameric as well as pentameric MscL, with the the ground-state configurations of sub-clusters of tetrameric and pentameric MscL approximately corresponding to $w = 9.5$ nm.

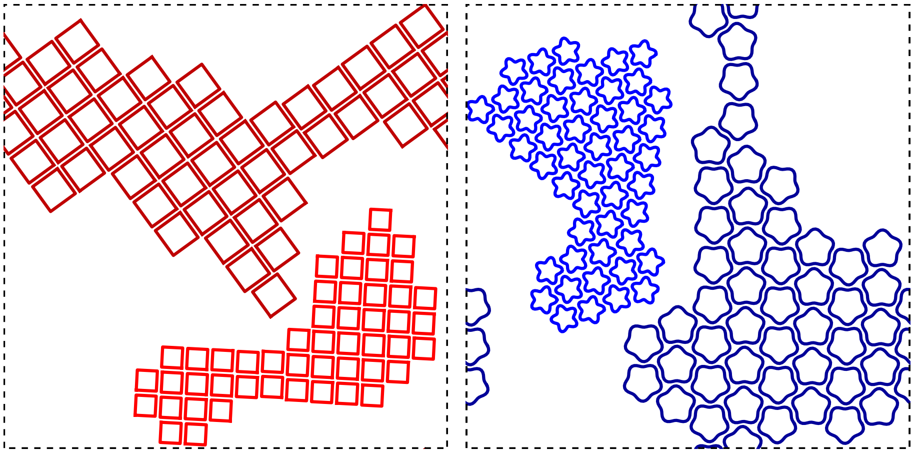

Figure S15: Partially activated MscL clusters segregate into sub-clusters of closed and open MscL. As in Fig. 6(b,c) of the main text, we considered 50 closed and 50 open MscL, and obtained minimum-energy configurations of tetrameric and pentameric MscL through Monte Carlo simulations of translational and rotational diffusion of MscL with simulated annealing of pair interaction potentials, but using different random seeds or initial conditions.

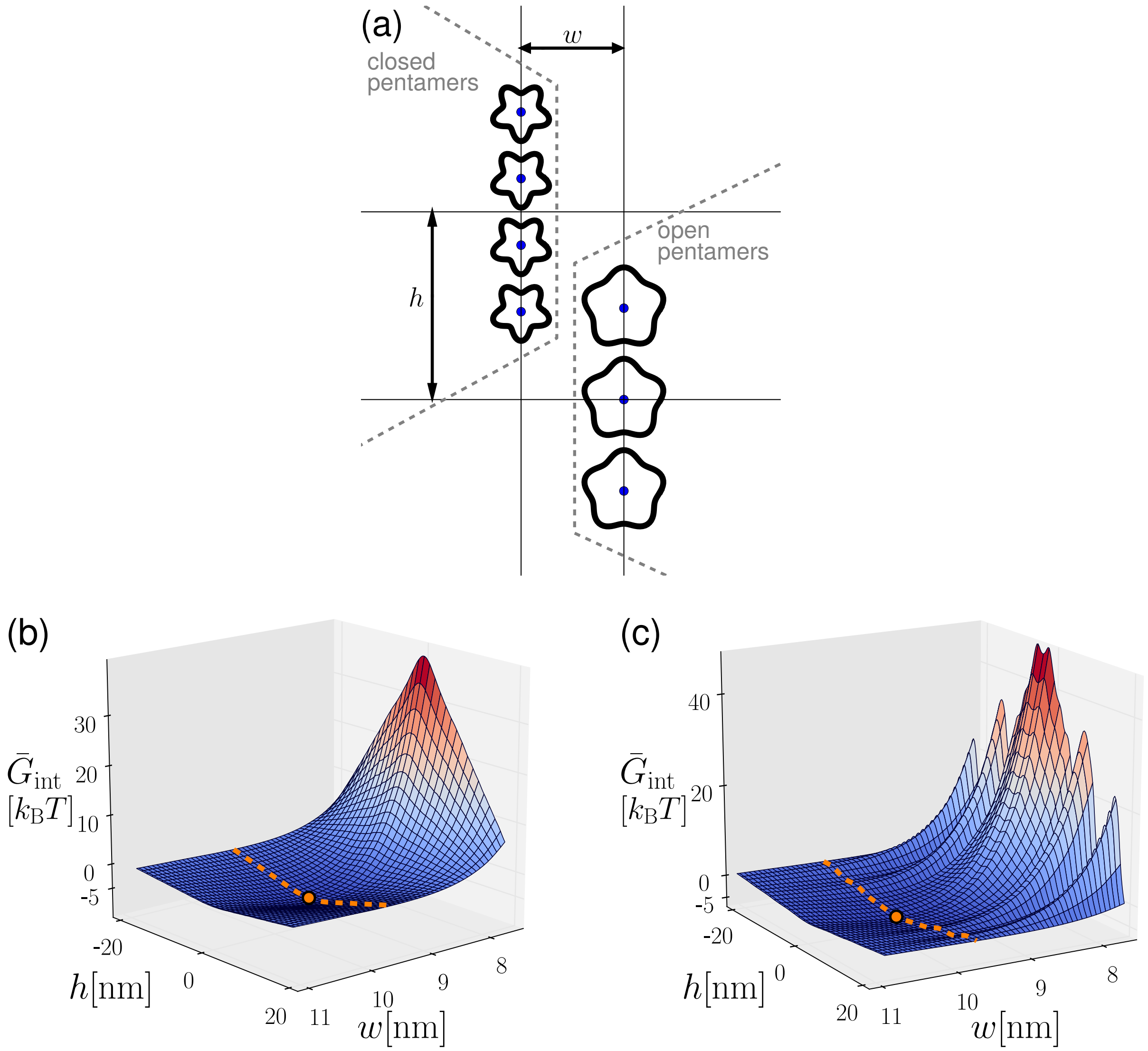

Figure S16: Bilayer thickness-mediated interactions between sub-clusters of closed and open MscL. We parameterize the total thickness-mediated interaction energy of sub-clusters of closed and open MscL, $\bar{G}_{\text{int}}$, by the coordinates (h, w) as illustrated in (a) for pentameric MscL. For our calculations we used four closed and three open MscL with face-on orientations of tetrameric MscL and the orientations of pentameric MscL indicated in (a). Total sub-cluster interaction energy, $\bar{G}_{\text{int}}$, for (b) tetrameric and (c) pentameric MscL. The minima of $\bar{G}_{\text{int}}$ (orange dots) are at $w \approx 9.5$ nm and $h \approx 0$, respectively.

S8 Supplementary Videos

Supplementary Video S1: Simulated annealing Monte Carlo simulation for a system composed of 100 closed tetrameric MscL.

Supplementary Video S2: Simulated annealing Monte Carlo simulation for a system composed of 100 closed pentameric MscL.

Supplementary Video S3: Simulated annealing Monte Carlo simulation for a system composed of 50 pentameric and 50 tetrameric MscL in the closed state.

Supplementary Video S4: Simulated annealing Monte Carlo simulation for a system composed of 50 closed and 50 open tetrameric MscL.

Supplementary Video S5: Simulated annealing Monte Carlo simulation for a system composed of 50 closed and 50 open pentameric MscL.

Supplementary Video S6: Simulated annealing Monte Carlo simulation for a system composed of 100 closed tetrameric MscL, with isotropically restricted minimum MscL separation $d_{\text{min}} = 6$ nm.

Supplementary Video S7: Simulated annealing Monte Carlo simulation for a system composed of 100 closed tetrameric MscL, with isotropically restricted minimum MscL separation $d_{\text{min}} = 8$ nm.

Supplementary Video S8: Simulated annealing Monte Carlo simulation for a system composed of 100 closed tetrameric MscL, with isotropically restricted minimum MscL separation $d_{\text{min}} = 7$ nm.

Supplementary Video S9: Simulated annealing Monte Carlo simulation for a system composed of 100 closed pentameric MscL, with isotropically restricted minimum MscL separation $d_{\text{min}} = 7$ nm.

References

[1] H. W. Huang. Deformation free energy of bilayer membrane and its effect on gramicidin channel lifetime. *Biophys. J.*, 50(6):1061, 1986.

[2] T. Ursell, K. C. Huang, E. Peterson, and R. Phillips. Cooperative gating and spatial organization of membrane proteins through elastic interactions. *PLoS Comput. Biol.*, 3(5):e81, 2007.

[3] R. Phillips, T. Ursell, P. Wiggins, and P. Sens. Emerging roles for lipids in shaping membrane-protein function. *Nature*, 459:379, 2009.

[4] T. Ursell et al. The role of lipid bilayer mechanics in mechanosensation. In A. Kamkin and I. Kiseleva, editors, *Mechanosensitivity in Cells and Tissues 1: Mechanosensitive Ion Channels*, pages 37–70. Springer Press, New York, 2008.

[5] P. Wiggins and R. Phillips. Analytic models for mechanotransduction: Gating a mechanosensitive channel. *Proc. Natl. Acad. Sci. U.S.A.*, 101(12):4071, 2004.

[6] P. Wiggins and R. Phillips. Membrane-protein interactions in mechanosensitive channels. *Biophys. J.*, 88(2):880, 2005.

[7] C. L. Henley. Sphere packings and local environments in Penrose tilings. *Phys. Rev. B*, 34:797, 1986.

[8] E. Luijten and J. Liu. Cluster algorithms: Beyond suppression of critical slowing down. *AIP Conference Proceedings*, 690(1):225, 2003.

[9] B. A. Camley and F. L. H. Brown. Diffusion of complex objects embedded in free and supported lipid bilayer membranes: role of shape anisotropy and leaflet structure. *Soft Matter*, 9:4767, 2013.

[10] S. L. Grage et al. Bilayer-mediated clustering and functional interaction of MscL channels. *Biophys. J.*, 100(5):1252, 2011.
